# Treatment of deep cavities using a perforator-based island flap with partial de-epithelization

**DOI:** 10.1186/s12893-018-0431-2

**Published:** 2018-11-12

**Authors:** Jung Woo Chang, Se Won Oh, Jeongseok Oh, M. Seung Suk Choi

**Affiliations:** 1 Department of Plastic and Reconstructive Surgery, Hanyang University Guri Hospital, Hanyang University College of Medicine, 249-1, Gyomun-dong, Guri-si, Gyeonggi-do 471-701 Korea; 2 Department of Plastic and Reconstructive Surgery, Hanyang University College of Medicine, 17 Haengdang-Dong, 133-792 Seongdong-Gu, Seoul, Korea

**Keywords:** Deep cavity, Pressure sore, Fistula, Perforator flap, De-epithelization

## Abstract

**Background:**

The perforator-based island flap is a popular option for defect coverage. In cases with deep cavities, however, the classical island flap may not be a suitable option. By de-epithelization of the peripheral portion of a perforator-based island flap, the distal part of the flap can be used to fill deep spaces, as the flap can be folded and inserted into the spaces.

**Methods:**

From June 2015 to April 2017, 21 cases of deep internal defects were reconstructed with perforator-based island flaps with peripheral de-epithelization. A fasciocutaneous flap was elevated and rotated with the pivot point on the perforator. After performing de-epithelization on the periphery of the flap, the de-epithelized portion of the flap was inserted and anchored into the internal defect. Demographic information about the patients, the size of the defects, the perforators that were used, and complications were recorded.

**Results:**

During the follow-up period (mean, 14.2 months) of total 21 cases, no major complications such as flap loss occurred. In 2 cases, a minor complication was observed. Temporary flap congestion was seen in 1 case, and was treated with a short period of leech therapy, and the other case was partial necrosis on the flap margin, which was cured with minimal debridement and conservative treatment. No major problems have occurred, especially on the de-epithelized part of the flap and in the occupied space.

**Conclusions:**

With performing careful procedure, a perforator-based island flap with partial de-epithelization can be a useful option for the surgical treatment of deep cavities.

**Trial registration:**

This study was retrospectively registered in the institutional review board on human subjects research and the ethics committee, Hanyang University Guri Hospital (Institutional Review Board File No. 2018–01–003-002 https://www.e-irb.com:3443/devlpg/nlpgS200.jsp).

## Background

Deep cavities are difficult wounds to treat. Generally, deep cavities have a cutaneous opening, and a space with considerable depth from the opening is present [1]. The direction of the space is variable, and can be horizontal, vertical or oblique [2]. Iceberg-type pressure sores and cutaneous fistulae are typical examples. To treat these deep cavity wounds, surgical methods are preferred, as conservative treatment such as negative pressure wound therapy requires a long period of treatment.

The perforator–based island flap is currently a popular surgical option for the treatment of defect wounds [3–7]. Especially in cases with pressure sore defects, it is the most popular option, as it can be transferred with less morbidity on the donor site [8–10]. However, the perforator-based island flap is usually a simple fasciocutaneous flap, meaning that it is not suitable for complex wound coverage. As deep cavities are complex wounds which easily remain seroma or bacterial colonization [11], the options for deep cavity treatment should also be complex. To fill the complex morphology of a deep cavity wound, the morphology of the flap must be modified [12].

The modification of the flap in this study, is peripheral de-epithelization of the perforator–based island flap. As the de-epithelized portion of the flap can be folded and inserted into the deep space, it can provide a cavity-occupying effect [13, 14].

## Methods

This study was conducted in conformity with the World Medical Association Declaration of Helsinki. From June 2015 to April 2017, 21 cases of deep cavities were treated using the modified perforator-based island flaps. Patient demographics, the size of the defect including the cavity, the perforators that were used, and postoperative complications, were recorded.

The operative procedure was performed under general anesthesia. For complete debridement of the affected tissues, all surface areas in the defect, including the deep cavity, were painted with gentian violet. The painted areas were completely excised to avoid recurrence after surgery. After the complete excision of the surface areas, perforator detection was performed with hand-held Doppler. When a healthy perforator was detected near the defect opening, the island flap was designed. The point where the perforator emerged was marked as the pivot point, and the distance from this point to the end of the deep cavity was measured. The flap was designed by applying the measured distance to the length from the pivot point to the end of the flap. The width of the flap was same as the width of the cavity, and the direction of the flap was decided by considering the arc of rotation and possibility of donor site closure. The ratio, length to width, was limited not to exceed 3:1, as a narrow flap can result in blood supply limit. The width of flap was also limited to 10 cm, for ensured primary closure on donor site. The arc of rotation should be less than 180° to prevent excessive twisting of the perforator.

After designing the flap, fasciocutaneous flap elevation was performed in a distal-to-proximal fashion. When dissecting near the pivot point, careful handling is needed to avoid perforator injury. Excessively fine dissection, such as skeletonization of the perforator should be avoided. After complete islanding of the flap, the flap was rotated toward the wound. When rotating the flap, there should be no tension on the perforator. If the flap can be rotated toward the wound without tension, the flap should be set according to the morphology of the wound by inserting the distal portion of the flap into the deep cavity. The inserted portion was then marked on the surface of the flap. The flap was taken out, and the marked portion was de-epithelized with the scalpel. The de-epithelized portion was inserted into the space again, and anchored with sutures to be fixed into the space. After confirming that the flap was positioned with the de-epithelized portion in the space and the intact portion on the cavity opening, both donor and flap sites were closed with sutures.

## Results

The 21 cases included in this study consisted of 16 pressure sores, 2 meningomyelocele defects, and 3 cutaneous fistulae (Table 1). There were 12 males and 9 females, and their mean age was 60.4 years (range, 31–81 years). Among the 21 cases, 9 superior gluteal artery perforators (SGAPs), 3 inferior gluteal artery perforators (IGAPs), 3 deep inferior epigastric artery perforators (DIEPs), 3 deep femoral artery perforators, and 3 tensor fascia lata perforator, were used as the pedicle of the island flap. During the follow-up period, which lasted for a mean of 14.2 months (range, 7–25 months), no major complications, such as flap loss, were observed.

**Table 1:** Patient demographics and characteristics of the flaps

No.	Age range (years)	Defect	Size, (cm^2^)	Depth of cavity (cm)	Size of de-epithelization (cm^2^)	Perforator	Follow-up (months)	Complication
1	70–79	Sacral sore	10 × 6	3	6 × 3	SGAP	25	None
2	80–89	Sacral sore	17 × 10	5	10 × 5	SGAP	20	None
3	60–69	Ischial sore	8 × 5	2	5 × 2	DFP	18	None
4	50–59	Meningomyelocele	10 × 4	4	5 × 4	SGAP	18	None
5	60–69	Sacral sore	15 × 13	3	10 × 3	SGAP	18	None
6	80–89	Sacral sore	11 × 7	4	6 × 4	IGAP	17	None
7	70–79	Ischial sore	12 × 10	3	8 × 3	DFP	15	None
8	60–69	Sacral sore	12 × 10	4	10 × 4	SGAP	15	None
9	50–59	Meningomyelocele	7 × 3	4	4 × 3	SGAP	15	None
10	30–39	Cutaneous fistula	4 × 4	9	9 × 4	DIEP	13	Congestion
11	30–39	Sacral sore	10 × 10	2	10 × 2	SGAP	13	None
12	30–39	Cutaneous fistula	4 × 4	9	9 × 4	DIEP	13	Partial necrosis
13	60–69	Trochanter sore	9 × 7	3	7 × 3	TFLP	13	None
14	50–59	Sacral sore	9 × 5	3	5 × 3	IGAP	13	None
15	60–69	Sacral sore	10 × 11	4	8 × 4	SGAP	12	None
16	40–49	Sacral sore	4 × 2	3	3 × 2	IGAP	12	None
17	40–49	Ischial sore	12 × 6	4	6 × 4	DFP	12	None
18	70–79	Cutaneous fistula	3 × 4	4	4 × 4	DIEP	10	None
19	80–89	Trochanter sore	8 × 6	2	6 × 2	TFLP	10	None
20	80–89	Trochanter sore	6 × 5	1	4 × 1	TFLP	10	None
21	60–69	Sacral	15 × 8	4	8 × 4	SGAP	7	None

Abbreviations: *SGAP* Superior gluteal artery perforator, *DFP* Deep femoral artery perforator, *IGAP* Inferior gluteal artery perforator, *DIEP* Deep inferior epigastric artery perforator, *TFLP* Tensor fascia lata perforator

In 2 cases (9.5%), a minor complication was observed. Temporary flap congestion after the operation was observed in 1 case. The congestion was observed on the second postoperative day, but it was treated with a short period of leech therapy. The other case involved partial necrosis at the flap margin. After observing the demarcation, it was cured with minimal debridement and conservative treatment. Of particular note, no problems occurred in the de-epithelized part of the flap and the space it occupied. No cavity-related complications like recurrence of infection, were observed. All cases with deep cavities were completely treated using the perforator-based island flap with partial de-epithelization.

### Case 1

**Fig. 1:**
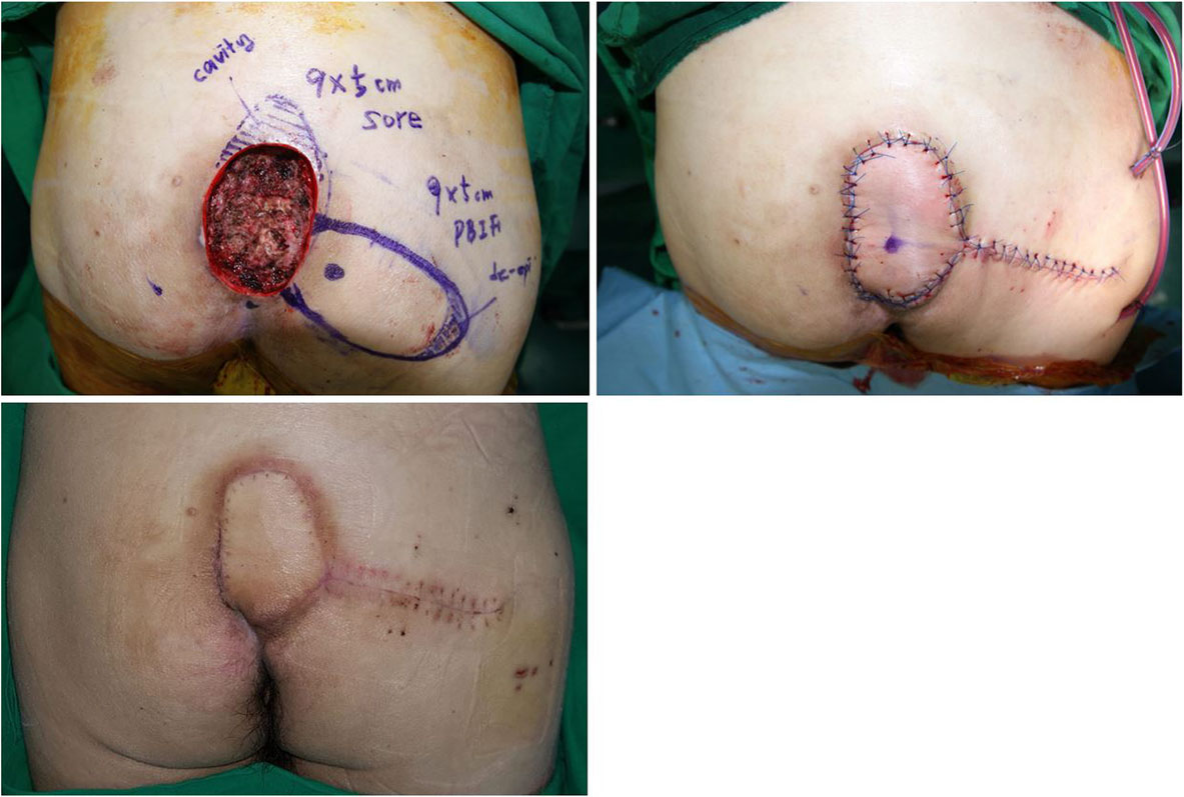
A patient (age range 50–59 years) with a stage IV sacral sore. (Upper left) After complete debridement, a 9 × 5 cm wound with a horizontally oriented cavity in cephalad, was seen. The marked area on the distal portion of the flap was de-epithelized. (Upper right) After transposing the flap and filling the cavity with the de-epithelized portion, both the recipient and the donor wounds were closed with sutures. (Below) The wound healed without any problems

A patient (age range 50–59 years) presented with a stage IV sacral sore. The patient was paraplegic, which led him to his chronic bedridden state. The wound measured 9 × 5 cm with its internal cavity. After complete debridement, a deep cavity was still present on cephalic side of the defect. An IGAP-based perforator flap was elevated to cover the defect, and de-epithelization was performed on the periphery to fill the cavity. After complete inset of the flap on the wound, both donor and recipient sites were closed primarily, with negative-suction drains inserted. No postoperative complications or recurrence took place during the follow-up period (Fig. 1).

### Case 2

**Fig. 2:**
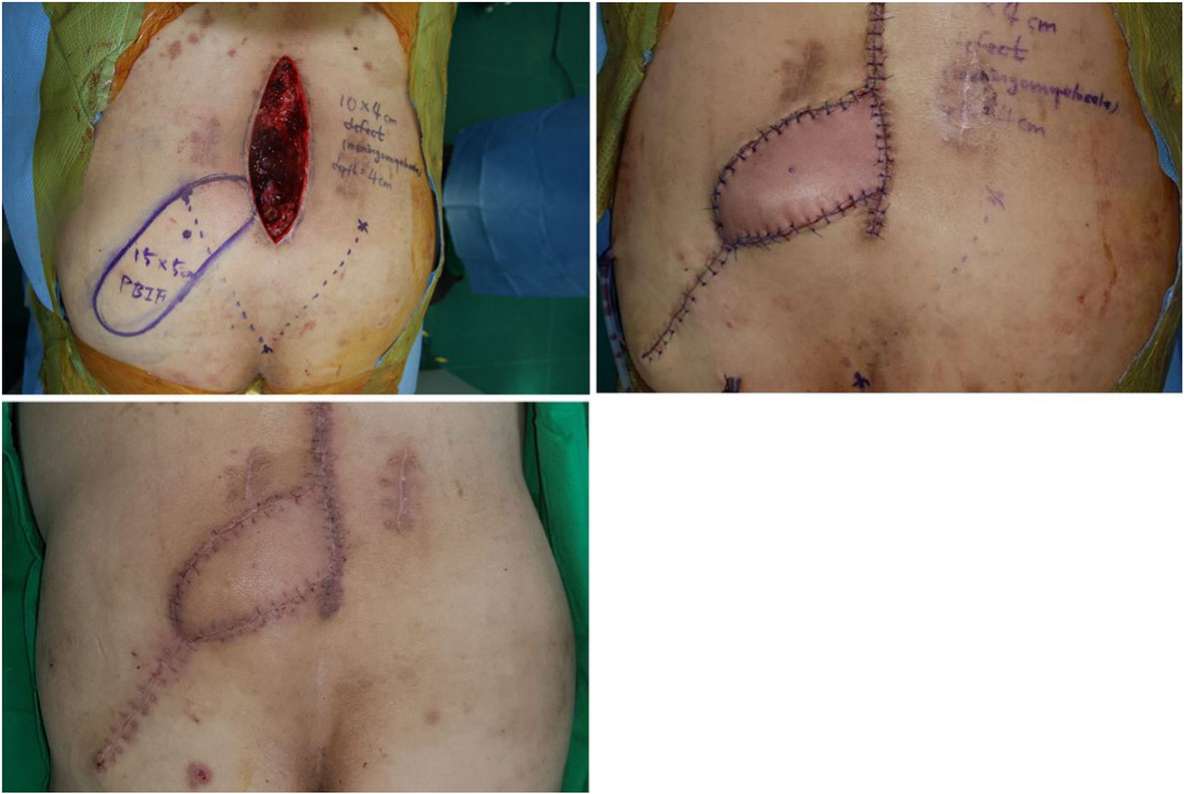
A patient (age range 50–59 years) with a meningomyelocele defect on the lumbar region. (Upper left) A 10 × 4 cm wound with a vertically oriented cavity, was observed after complete excision of the meningomyelocele. The distal half of the flap was de-epithelized, and inserted into the cavity. (Upper right) After complete inset of the flap, both the recipient and the donor wounds were closed with sutures. (Below) The wound healed without any problems

A patient (age range 50–59 years) presented with a meningomyelocele defect in the lumbar region. The patient had undergone surgery twice in another clinic. As the patient suffered from recurrent meningomyelocele, flap coverage was needed immediately after complete excision of the recurrent mass. The size of the defect after the complete excision, was 10 × 4 cm, and the depth of the cavity was 4 cm. To cover the vertically oriented cavity, an SGAP-based island flap with peripheral de-epithelization was elevated. The de-epithelized portion of the flap completely filled the cavity, and no complications or recurrence were observed during the follow-up period (Fig. 2).

### Case 3

**Fig. 3:**
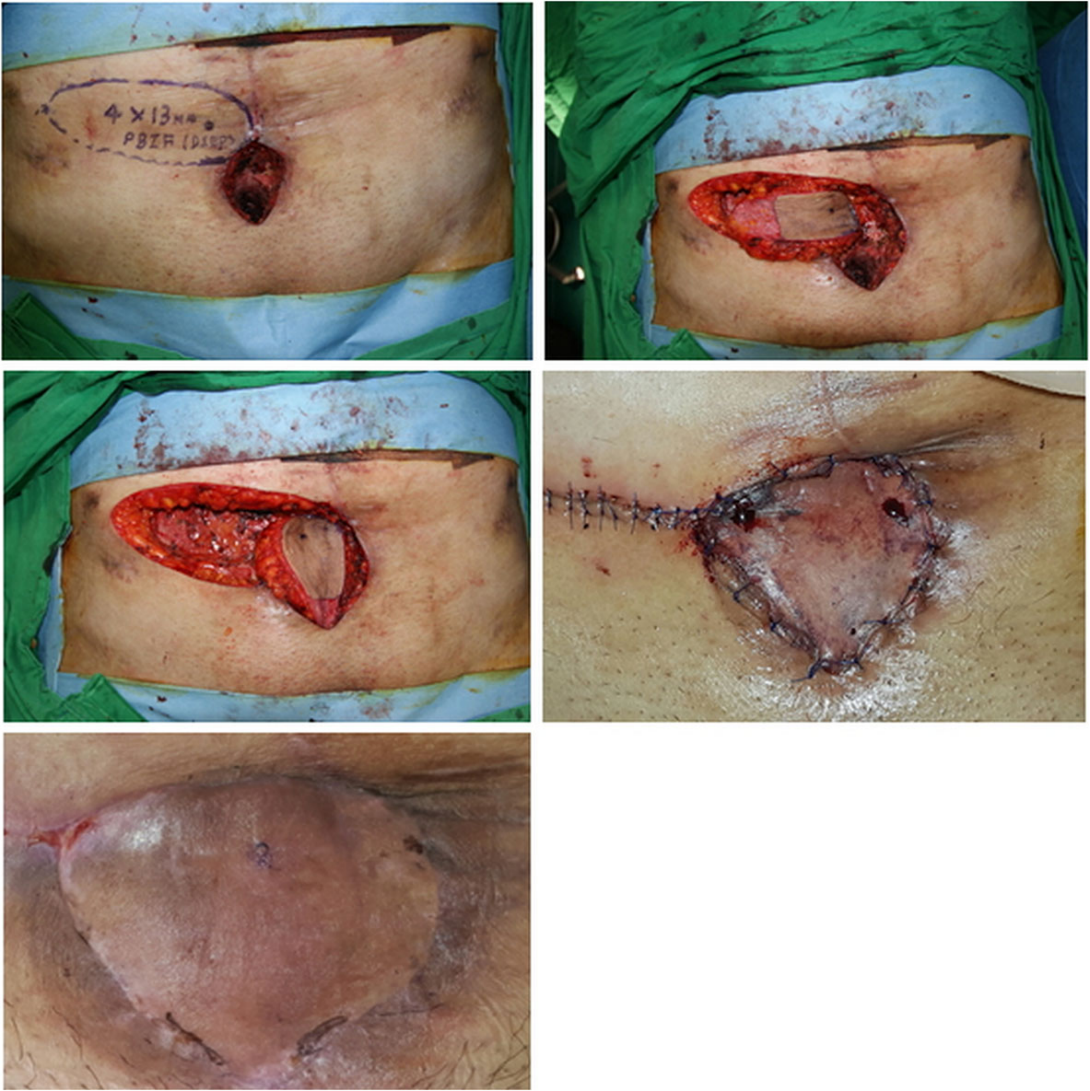
A patient (age range 30–39 years) with a cutaneous fistula on the lower abdomen. (Upper left) A 4 × 4 cm cutaneous opening with a 9 cm length deep cavity obliquely oriented beneath the pelvic bone, was seen. An island flap was designed near the wound. (Upper right) The portion that would be inserted into the cavity, was de-epithelized. (Center left) The flap was transposed with its intact portion on the opening of the wound, while the de-epithelized portion was placed into the cavity. (Center right) The flap showed temporary congestion, and leech therapy was applied for a short duration. (Below) The wound healed without any problems

A patient (age range 30–39 years) presented with a cutaneous fistula on the lower abdomen. The patient had experienced a severe pelvic bone fracture and bladder rupture, which resulted in the formation of massive adhesion tissues between the pelvic bone and bladder. The cutaneous fistula was formed in the adhesion tissue. The size of the fistula was found to be 4 × 4 cm of opening and 9 cm length of tract after a complete fistulectomy. The cavity started from the cutaneous opening, and the direction was oblique to connect with the anterior portion of the bladder. After reconstructing the bladder opening by a urologic surgeon, a DIEP-based island flap with peripheral de-epithelization was elevated to fill the obliquely oriented cavity. The flap showed temporary congestion, but the congestion was straightforwardly controlled with a short period of leech therapy (Fig. 3).

## Discussion

Deep cavity wounds are difficult to treat, because they are usually vulnerable to infection which prolongs healing time [15, 16]. Incomplete treatment of dead space within the wound often induces recurrence and chronicity. The rapid and complete solution for deep cavity wound is obliterating dead space with well vascularized tissue through surgical intervention [17, 18]. To provide obliteration of dead space, using muscle flap around the wound can be considered at first [19–24]. However, using muscle flap leaves a potential of huge donor site morbidity, and requires additional skin flap to cover the cutaneous opening of the wound.

Since the perforator-based island flap was introduced by Koshima et al. [25] in 1993, it has been widely used for defect coverage. When comparing the perforator-based island flap with the musculocutaneous local flap introduced by Mathes and Nahai in 1979 [ 26], its advantages are, low donor site morbidity and the possibility of future reconstruction in cases of recurrence. As perforator-based island flap preserves underlying muscle, its donor site morbidity is lower than that of musculocutaneous flap which sacrifices both skin and muscle layer. Furthermore, preserved muscle contains additional perforators which enable future reconstruction with another perforator-based island flap in cases of recurrence. These advantages led this option to become popular. However, the classical form of the perforator-based island flap is not perfect for deep cavity filling. Deep cavity wounds, such as fistulae and iceberg-type pressure sores, typically have morphology with a deep internal space combined with a cave-like cutaneous opening. The classical island flap can cover only the cutaneous opening, but not the internal space. However, if the skin layer of the island flap is removed, it can be inserted into this space [27–30]. The modification of the flap in this study, was peripheral de-epithelization. The proximal portion with an intact skin layer was placed on cutaneous opening of the wound, and the distal portion that underwent de-epithelization was folded and inserted into the deep space to fill the cavity. As the proximal portion with the intact skin layer was exposed on the outside, it served as a monitoring flap. Since the distal portion, which is buried in the deep space, cannot be monitored directly, its circulatory condition should be assessed based on the monitoring flap.

**Fig. 4:**
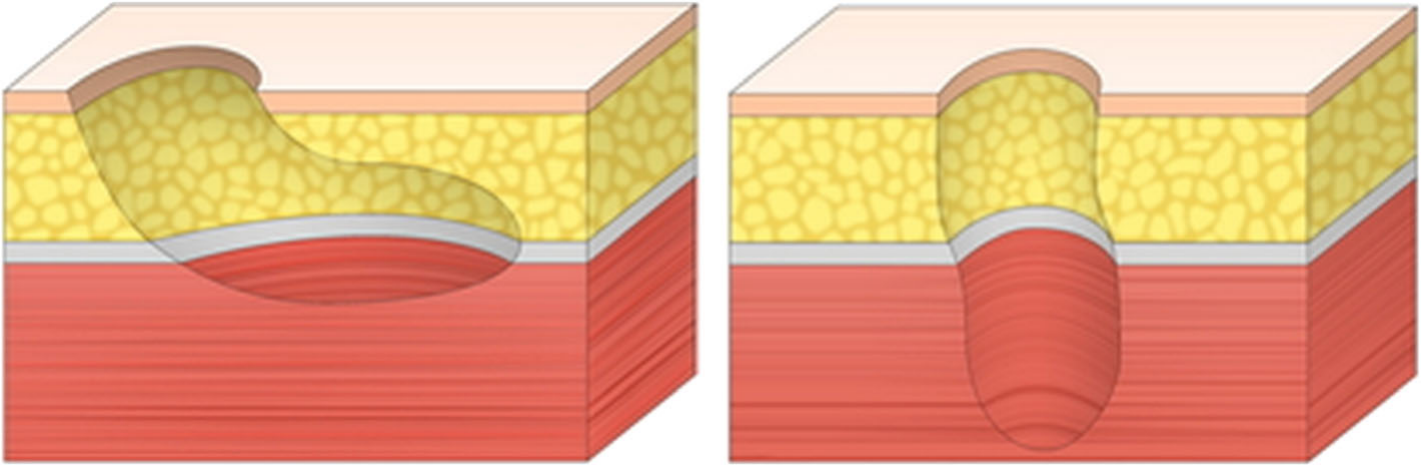
Classification of deep cavity wounds according to their orientation. (Left) Schematic image of type I wounds, with a horizontally oriented cavity. (Right) Schematic image of type II wounds, with a vertically or obliquely oriented cavity

Deep cavity wounds can be classified into 2 types by their orientation (Fig. 4). The first type is a horizontally oriented cavity. A typical example of this type is an iceberg-shaped pressure sores. The direction of the deep space is horizontal, and its roof is usually covered with a skin layer. If the classical perforator-based island flap is used to reconstruct this type of wound, the skin and soft tissue layer covering the roof of the cavity should be excised. Excision of the healthy skin layer of the wound is tissue-wasting. However, using the modified form of the perforator-based island flap described in this study provides the possibility of saving the tissue on the wound. The second type involves a vertically or obliquely oriented cavity. This type includes cutaneous fistulae and meningomyelocele defects. As the direction of the deep space is vertical or oblique, the de-epithelized portion of the flap should be folded more. The proximal intact portion is usually placed horizontally on the opening of the wound, but the distal de-epithelized portion should be placed vertically or obliquely in the space. For this reason, the flap in the second type, sometimes, needs to be folded excessively, whereas the flap in the first type only requires minimal folding. As excessive folding of the flap can lead to circulatory problems in the flap, reconstruction of the second type wound requires more intensive care.

In this study, 2 circulatory problems were observed. They were venous congestion and marginal necrosis on the proximal portion, which plays a role as a monitoring flap. These problems, fortunately, were solved with conservative treatment and did not affect the distally buried component of the flap. In these 2 cases, a perforator that may have been injured, was used. In fact, these 2 problem cases belonged to the same patient, who previously underwent pelvic bone injury and bladder rupture. When the first fistulectomy and flap surgery were performed, the patient experienced temporary congestion on the flap, but it was cured completely. After complete healing, the patient experienced another event of bladder rupture, which resulted in the formation of new fistula on the other site. As the authors did not notice that the problem in the first operation resulted from using an injured deep inferior epigastric artery perforator, another similar perforator near the first flap was used for the second surgery. This time, the patient experienced marginal necrosis on the flap. After observing 2 problems on the same patient, the authors concluded that the perforators used in both operations may have been injured when the patient experienced pelvic bone fracture, resulting in circulatory problems in the flaps. If the cause of the problem has been noticed at first, another flap such as a distant pedicled flap or a free flap, would have been selected for the second operation rather than an island flap with an injured perforator.

## Conclusions

The perforator-based island flap with partial de-epithelization is a modified flap for the treatment of deep cavity wounds with maintaining the benefits of classical perforator-based island flaps. However, the weak point of this flap is that the de-epithelized portion of the flap, which may be most vulnerable in terms of circulation, cannot be monitored directly, as the de-epithelized portion is buried in the cavity. For this reason, the factors that can result in poor circulatory conditions on the flap should be avoided. Excessive folding of the flap is dangerous. In trauma cases, using injured perforators from the zone of the injury also should be avoided. With careful procedures, the flap can be transferred without anxiety regarding the buried portion. As it has been proved in this study, the perforator-based island flap with partial de-epithelization is a useful option for the treatment of deep cavity wounds.

## Abbreviations

DFP: Deep femoral artery perforator
DIEP: Deep inferior epigastric artery perforator
IGAP: Inferior gluteal artery perforator
SGAP: Superior gluteal artery perforator
TFLP: Tensor fascia lata perforator
